# MCL-1 is a prognostic indicator and drug target in breast cancer

**DOI:** 10.1038/s41419-017-0035-2

**Published:** 2018-01-16

**Authors:** Kirsteen J. Campbell, Sandeep Dhayade, Nicola Ferrari, Andrew H. Sims, Emma Johnson, Susan M. Mason, Ashley Dickson, Kevin M. Ryan, Gabriela Kalna, Joanne Edwards, Stephen W. G. Tait, Karen Blyth

**Affiliations:** 1 CRUK Beatson Institute, Garscube Estate, Switchback Road, Bearsden, Glasgow, G61 1BD UK; 2 Institute of Cancer Sciences, University of Glasgow, Glasgow, G61 1QH UK; 3 Tumour Microenvironment Team, The Institute of Cancer Research, Chester Beatty Laboratories, London, SW3 6JB UK; 4 Applied Bioinformatics of Cancer, University of Edinburgh Cancer Research Centre, Institute of Genetics and Molecular Medicine, Edinburgh, EH4 2XR UK

## Abstract

Analysis of publicly available genomic and gene expression data demonstrates that *MCL1* expression is frequently elevated in breast cancer. Distinct from other pro-survival Bcl-2 family members, the short half-life of MCL-1 protein led us to investigate MCL-1 protein expression in a breast cancer tissue microarray and correlate this with clinical data. Here, we report associations between high MCL-1 and poor prognosis in specific subtypes of breast cancer including triple-negative breast cancer, an aggressive form that lacks targeted treatment options. Deletion of MCL-1 in the mammary epithelium of genetically engineered mice revealed an absolute requirement for MCL-1 in breast tumorigenesis. The clinical applicability of these findings was tested through a combination of approaches including knock-down or inhibition of MCL-1 to show triple-negative breast cancer cell line dependence on MCL-1 *in vitro* and *in vivo*. Our data demonstrate that high MCL-1 protein expression is associated with poor outcome in breast cancer and support the therapeutic targeting of MCL-1 in this disease.

## Introduction

Breast cancer survival has increased in recent decades due, in part, to the introduction of targeted therapies. Development of these therapeutics has arisen from an increased understanding of the diverse molecular characteristics of breast tumours such as expression of receptors for oestrogen, progesterone or amplification of human epidermal growth factor receptor 2 (HER2). For example, hormonal therapies such as Tamoxifen have efficacy in breast cancers expressing the oestrogen receptor (ER) while cancers with *ERBB2* amplification (HER2) can be treated with HER2 targeting therapies such as trastuzumab (e.g., Herceptin). However, resistance to conventional cytotoxic drugs and to new targeted therapies can emerge and despite dramatic improvements in patient outcome, breast cancer remains the leading cause of cancer mortality worldwide in females^1^.

Evasion of apoptosis promotes tumour development and also acts as a barrier to cancer therapy-induced cell death. Mitochondrial-dependent apoptosis is controlled by Bcl-2 family members—these proteins control cell fate by regulating mitochondrial integrity. During apoptosis, upregulation of pro-apoptotic Bcl-2 members such as BIM (so called BH3-only proteins) overwhelms anti-apoptotic Bcl-2 function and activates BAX/BAK triggering mitochondrial outer membrane permeabilisation and cell death^2^. Aberrant increases in the level of anti-apoptotic Bcl-2 proteins such as BCL-2, MCL-1 or BCL-XL prevents apoptosis, this both promotes cancer and allows resistance to cancer therapy-induced cell killing^3^.

Recent progress has been made in the development of inhibitors of anti-apoptotic BCL-2 proteins with the aim of restoring apoptosis in cancer^4^. Small molecules have been developed, called BH3-mimetics that functionally mimic BH3-only proteins, freeing pro-apoptotic Bcl-2 proteins to trigger or sensitize to cell death. The value of such drugs has been highlighted in the treatment of haematological malignancies where the BCL-2 targeting drug venetoclax has recently secured FDA approval for use in some types of chronic lymphocytic leukaemia^5,6^. Due to differential binding affinities, various BH3-mimetics display specificity for particular anti-apoptotic BCL-2 proteins. BH3-mimetics targeting BCL-2/BCL-XL have also shown promise in preclinical studies of solid tumours, including breast, when used in combination with docetaxel or tamoxifen^7,8^ but resistance can be mediated by MCL-1^9,10^. In addition to differential BH3-binding properties, MCL-1 is distinguished by its short protein half-life and ability to regulate mitochondrial metabolism^11,12^. There has been intense activity to develop BH3-mimetics to target MCL-1 with recent progress; A1210477 shows impressive anti-cancer effects *in vitro* on diverse cancer cell lines^13,14^; UMI-77 is effective as a single agent on pancreatic cancer cell lines *in vitro* and in xenograft models^15^; and S63845 shows tumour-specific cell killing in leukaemia, lymphoma and myeloma in a variety of *in vitro*, xenograft and genetically modified mouse models^16^. Encouragingly, tumour cells seem particularly sensitive to MCL-1 inhibition suggesting an adequate therapeutic window.

As well as playing a role in resistance to therapy, elevated MCL-1 can actually drive haematopoietic tumour development^17^. This oncogenic role for MCL-1 may be widespread as the *MCL1* locus is one of the most frequently amplified regions of the human genome across a wide variety of cancers including breast cancer^18^. Recent evidence from *in vitro* experiments suggests an important role for MCL-1 in breast cancer cell survival^10,19,20^, particularly in triple-negative (TN) breast cancers^21–23^ and expression of a mutant form of BIM that specifically interacts with MCL-1 inhibits metastases of TN breast cancer cell lines in xenograft models^24^. TN breast cancers are aggressive with poor patient prognosis and because they lack expression of the ER and the progesterone receptor (PR) and do not have amplification of *ERBB2*, they do not respond to current targeted therapies.

There is a need for new therapeutic options to reduce the mortality burden of breast cancer. Given the emergence of BH3-mimetic drugs capable of targeting MCL-1 we investigated the expression and functional requirement for MCL-1 in breast cancer, systematically testing this through a combination of human breast tumour tissue analysis with correlation to clinicopathological data; breast cancer cell line testing *in vitro* and *in vivo*; and for the first time show a role for MCL-1 in mammary tumorigenesis using a genetically engineered mouse model.

## Results

### High MCL-1 protein expression predicts poor outcome in breast cancer

**Fig. 1:**
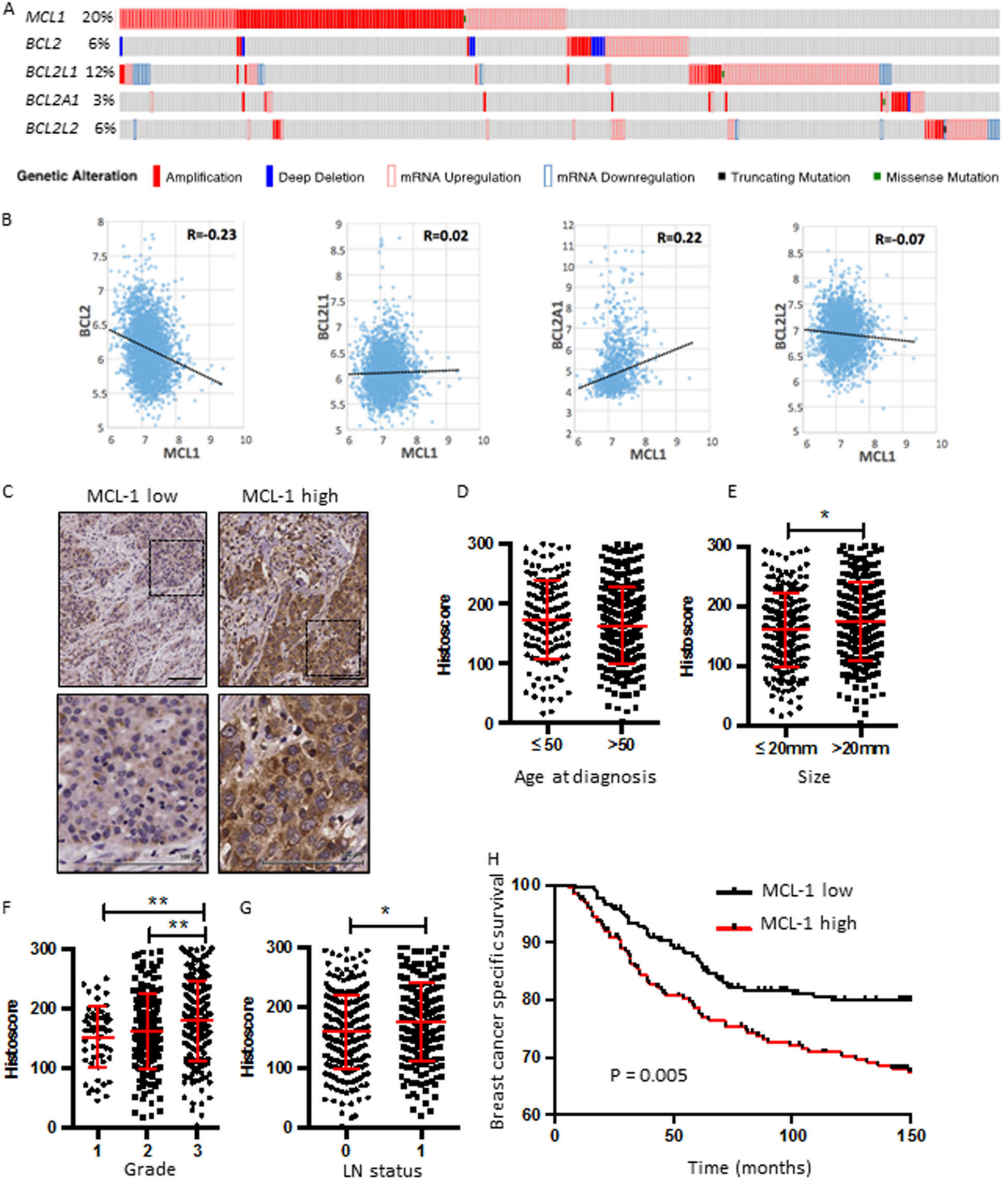
High levels of MCL-1 protein predict poor outcome in breast cancer **a** cBioportal analysis of pro-survival Bcl-2 family member gDNA and mRNA level using^25^ TCGA Breast Invasive Carcinoma data set. Unaltered cases not shown, *n* = 816 patients. The results shown in Fig. 1a are in the whole based upon data generated by the TCGA Research Network: http://cancergenome.nih.gov/. **b** Scatterplots of *MCL1* mRNA expression vs. *BCL2, BCL2L1* (BCL-xL), *BCL2A1* and *BCL2L2* (BCL-W) in combined Affymetrix data set of 2999 breast tumours^29^ *P* < 0.05; R > 0.03. **c** Representative MCL-1 immunohistochemistry images on primary operable breast tumour tissue microarray cores depicting low epithelium MCL-1 staining (left panels) and MCL-1 high (right panels). Scale bar = 100 μm, boxed areas in upper panels are shown at higher magnification in lower panels. **d-g** Comparison of MCL-1 protein levels in patient cohort by **d** age at diagnosis, **e** tumour size in mm, **f** tumour grade, **g** lymph node (LN) status where 0 = no tumour detected in lymph nodes and 1 = tumour detected in at least 1 lymph node. Each point represents the average MCL-1 histoscore of an individual patient from 2–3 independent biopsy cores and bars indicate mean ± SD * *P* < 0.05, **P < 0.01, Pearson Chi-Square test. **h** Kaplan–Meier survival plot of breast cancer-specific survival by MCL-1 protein level determined by histoscore, *n* = 420 patients, *P* = 0.005 Log-rank (Mantel-Cox) test

As amplification of the *MCL1* locus is frequently observed in a range of cancer types^18^ we investigated the frequency of elevated *MCL1* in breast cancer. Analysis of comprehensive, publically available data reveals that *MCL1* gene amplification and/or mRNA upregulation in breast cancers is at a frequency of up to 20% across different studies, in contrast to much lower frequency alteration of other pro-apoptotic Bcl-2 relatives (Fig. 1a The Cancer Genome Atlas (TCGA) Breast data^25–27^ and METABRIC data^28^ (not shown)). Of note, while increased *MCL1* was evident, both up- and downregulation of other family members *BCL2*, *BCL2L1(*BCL-XL), *BCL2A1*(A1) and *BCL2L2*(*BCL-*W) were observed. These data suggest an exquisite role for upregulated *MCL1* in breast cancer. Interestingly, *MCL1* mRNA levels were found to inversely correlate with *BCL2* (Fig. 1b and Supplementary Fig. 1) upon analysis of two large independent breast cancer data sets^26,27,29^. Positive correlation was observed between *MCL1* and *BCL2A1* mRNA while correlations with other pro-survival Bcl-2 proteins were not consistent between data sets (Fig. 1b and Supplementary Fig. 1). Unlike the relatively stable proteins BCL-2 and BCL-XL, MCL-1 has a very short half-life under normal conditions and thus a functional role for elevated MCL-1 may further manifest at the protein level. We, therefore, analysed MCL-1 protein expression by immunohistochemistry in a large tumour tissue microarray of 428 patients with primary operable breast cancer, and correlated MCL-1 expression with associated clinicopathological data (see Table 1 ^30^). MCL-1 expression was detected in almost every tumour. Using a weighted histoscore method, which captures intensity of staining as well as percentage of cell positivity^31^, a broad range of MCL-1 protein level in tumour epithelium was observed in different patient samples (Fig. 1c). While no correlation was observed between MCL-1 protein level and age of patient at diagnosis (Fig. 1d), we discovered a statistically significant shift in MCL-1 with increased tumour size, invasive grade and in cases where tumour had spread to lymph nodes (Fig. 1e-g, *P ≤ 0.05, **P ≤ 0.01 Pearson Chi-Square test). Consistent with these findings, high MCL-1 protein expression was found to correlate with poor patient prognosis *P* = 0.005 (Log-rank Mantel-Cox) (Fig. 1h).

**Table 1:** Clinicopathological characteristics of patients with primary operable breast cancer

Clinicopathological characteristics (total)	Patients (*n*%)
Age (≤50/≥51 years) (*n*=428)	141 (33%)/287 (67%)
Size (mm ≤20/>20) (*n*=427)	212 (50%)/215 (50%)
Tumour type (special type/lobular/ductal) (*n*=428)	24 (6%)/23 (5%)/381 (89%)
Grade (I/II/III) (*n*=426)	55 (13%)/176 (41%)/195 (46%)
Involved lymph node (Negative/positive) (*n*=420)	231 (55%)/189 (45%)
Oestrogen receptor status (ER-/ER+) (*n*=428)	182 (43%)/246 (57%)
Progesterone receptor status (PR-/PR+) (*n*=428)	259 (61%)/169 (39%)
ERBB2 amplification status (ERBB2-/ERBB2+) (*n*=426)	336 (78%)/90 (21%)
TN status (ER-,PR-,ERBB2-/ER+,PR+,ERBB2+) (426)	118 (28%)/308 (72%)
MCL-1 (High/low) (*n*=428)	220 (51%)/208 (49%)

### MCL-1 protein expression is important within specific breast cancer subtypes

**Fig. 2:**
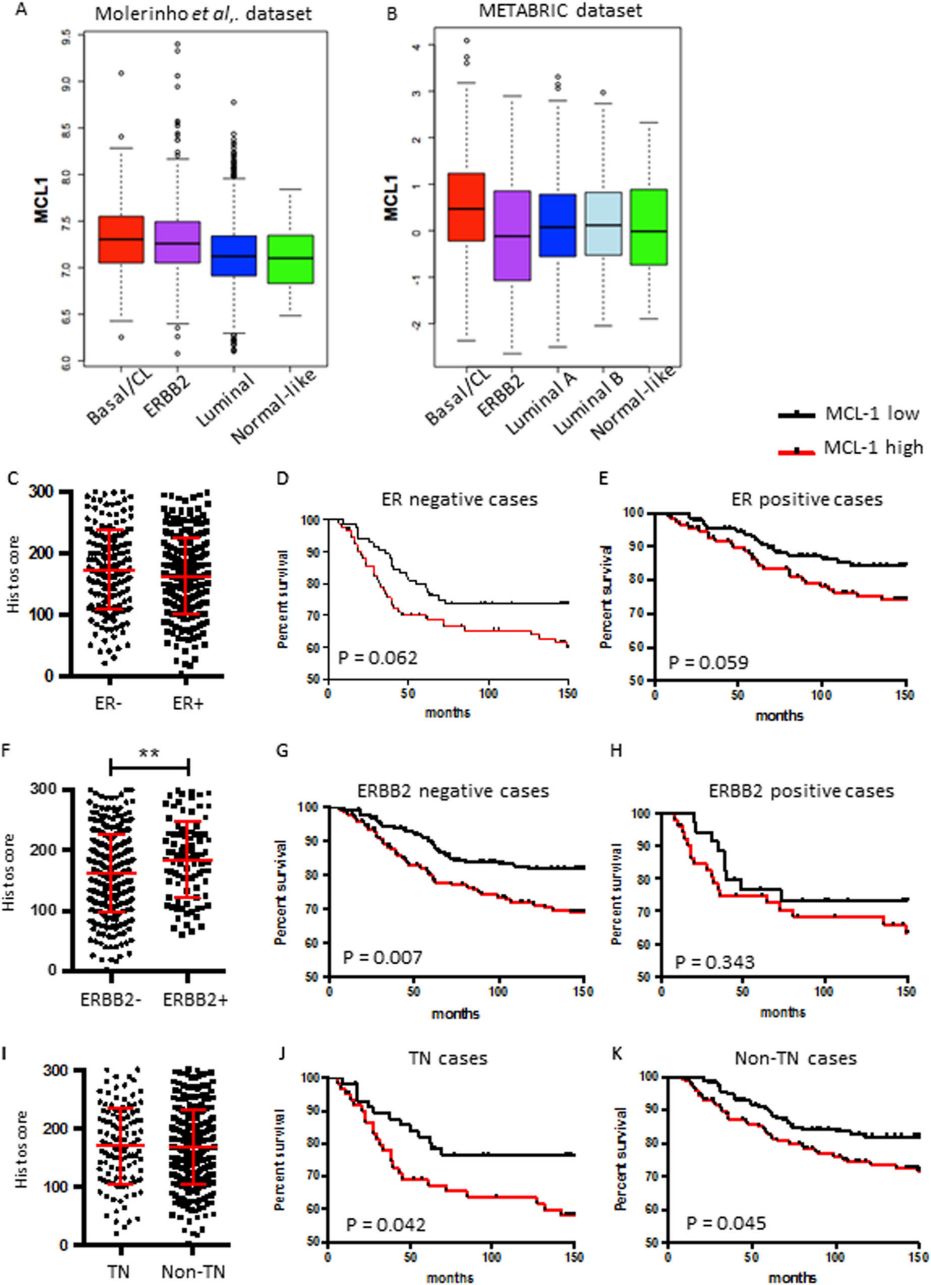
MCL-1 level is important within specific breast cancer subtypes **a** Comparison of *MCL1* mRNA expression across 2999 breast tumours^29^ segregated into subtypes^56^, Basal/CL (claudin low) *n* = 296, ERBB2 *n* = 716, Luminal *n* = 1959 and Normal-like, *n* = 28, Wilcoxon test Basal/CL v rest *P* = 7e-10. **b** Comparison of *MCL1* mRNA expression across 1904 breast tumours^28^ segregated into PAM50 + CL subtypes, Basal/CL *n* = 398, ERBB2 *n* = 220, Luminal A *n* = 697, Luminal B *n* = 461, Normal-like *n* = 140, Wilcoxon test Basal/CL v rest *P* = 6e-11. **c–k** Comparison of MCL-1 protein levels in patient cohort by **c–e** ER status, *n* = 181 ER negative, *n* = 246 ER positive; **f–h** ERBB2 status, *n* = 335 ERBB2 negative, *n* = 90 ERBB2 positive; **i–k** Triple-negative (TN) status, *n* = 118 TN, *n* = 308 non-TN. For **c**, **f**,** i** each point represents the average MCL-1 histoscore of an individual patient from 2–3 independent biopsy cores and bars indicate mean ± SD **P < 0.01, unpaired *t*-test. For Kaplan–Meier graphs, data are plotted for patients where follow-up data were available. Kaplan–Meier survival plots of breast cancer-specific survival segregated by MCL-1 protein level are shown **d**, **e**, **g**, **h**, **j**, **k** and *P*-values indicated on plots, Log-rank (Mantel-Cox) test. Black line indicates MCL-1 low cases and red line MCL-1 high cases

*MCL1* mRNA is higher in Basal (including Claudin-low (CL)) breast cancers relative to other subtypes (Fig. 2a, b) and we reasoned that MCL-1 may have differential prognostic significance in certain subtypes of breast cancer. Although within our TMA a similar range in MCL-1 protein level was apparent across ER-negative and ER-positive breast cancers, MCL-1 high cases in both groups appeared to have poorer prognosis than MCL-1 low cases (Fig. 2c-e). MCL-1 protein was significantly elevated in *ERBB2* amplified (ERBB2 positive) breast cancers (Fig. 2f) but intriguingly, did not significantly correlate with prognosis (Fig. 2h) and rather, exclusion of ERBB2-positive cases potentiated the association between high MCL-1 and poor prognosis *P* = 0.007 (Log-rank Mantel-Cox test), with 10 year survival now being stratified from 82 to 71% (Fig. 2g). Triple-negative (TN; i.e., ER/PR/ERBB2 negative) breast cancers are considered among the most aggressive of breast cancers and have no targeted treatment options. Although MCL-1 protein levels were comparable between TN and non-TN subtypes (Fig. 2i), MCL-1 high TN breast cancer patients showed the worst overall prognosis of all, *P* = 0.042 (Log-rank Mantel-Cox test) (Fig. 2j, k) with only 64% survival at 10 years vs. 77% for MCL-1 low TN cases. These findings emphasise the prognostic importance of MCL-1 protein expression in breast cancer.

### MCL-1 is required for breast cancer cell-line survival *in vitro*

**Fig. 3:**
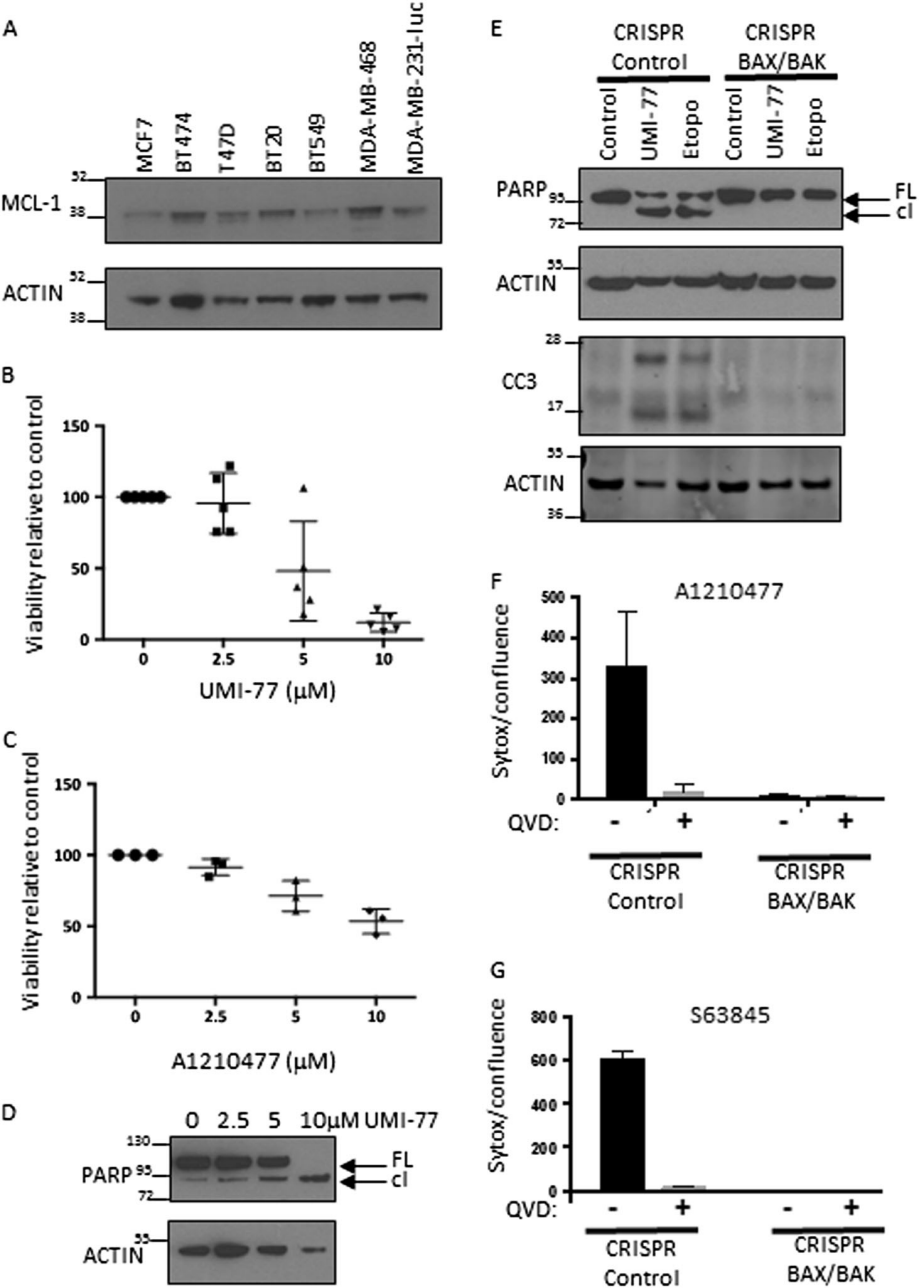
MCL-1 is required for breast cancer cell line survival *in vitro* **a** Western blot analysis of MCL-1 protein expression across a panel of human breast cancer cell lines. **b**, **c** MTS assay showing viability of MDA-MB-468 cells after 48 h treatment with indicated dose of MCL-1 inhibitor (**b**) UMI-77 or (**c**) A1210477. Bars indicate mean ± SD, of *n* = 3–5 independent experiments plated in triplicate. **d** Western blot showing full length (FL) and cleaved (cl) PARP following incubation of MDA-MB-468 cells with indicated doses of UMI-77 for 24 h. Actin as loading control shown below. **e** Western blot analysis (as for **d**) showing PARP cleavage and active caspase 3 (CC3) in MDA-MB-468 CRISPR/Cas9 edited for BAX/BAK deletion (see Supplementary Fig. S2B) or non-targeting control following 24 h treatment with 10 μM UMI-77 or 10 μM etoposide (etopo). Actin loading control is given for each membrane. **f**, **g** Incucyte Sytox Green cell death assay of cell lines described in **e** following 48 h treatment with 5 μM A1210477 (**f**) and 0.1 μM S63845 (**g**) in the presence or absence of 10 μM Q-VD-OPh caspase inhibitor. Cell death was calculated with the formula CD^treatment^-CD^basal^ where CD^treatment^ is Sytox Green cells/cell confluence following 48 h treatment with MCL-1 inhibitor and CD^basal^ is Sytox Green cells/cell confluence in control samples at 48 h. Graph represents mean ± SEM from *n* = 3–4 independent experiments plated in triplicate

MCL-1 protein expression has been shown in a wide range of breast cancer cell lines^23,24^. In agreement with this, we detected MCL-1 in cell lines representing the main subtypes of breast cancer (Fig. 3a). MDA-MB-468 cells were selected for further study as they expressed relatively high levels of MCL-1 and as a TN breast cancer cell line they are representative of a disease subtype that is in need of new therapies. Consistent with previous studies^22–24^ we found that MDA-MB-468 cells depend on MCL-1 for survival *in vitro* as the MCL-1 targeted BH3-mimetic inhibitors UMI-77 (Fig. 3b) and A1210477 (Fig. 3c) reduce viability of MDA-MB-468 cells in a dose-dependent manner. Sensitivity to MCL-1 inhibition was also observed in the ER-positive cell line MCF-7, although only at a higher dose (10 μM) (Supplementary Fig. S2A). Importantly, we find that this results from on-target effects of these drugs through engagement of the intrinsic apoptotic pathway as evidenced by induction of PARP cleavage (Fig. 3d) and caspase 3 activation that requires the presence of BAX/BAK (Fig. 3e and Supplementary Fig. S2B). Furthermore, MCL-1 inhibitor mediated cell death was abrogated by caspase inhibition with Q-VD-OPh (Fig. 3f, g) again showing on-target impact of these BH3-mimetics in breast cancer cells.

### Targeting MCL-1 restricts growth of TN breast cancer xenografts

**Fig. 4:**
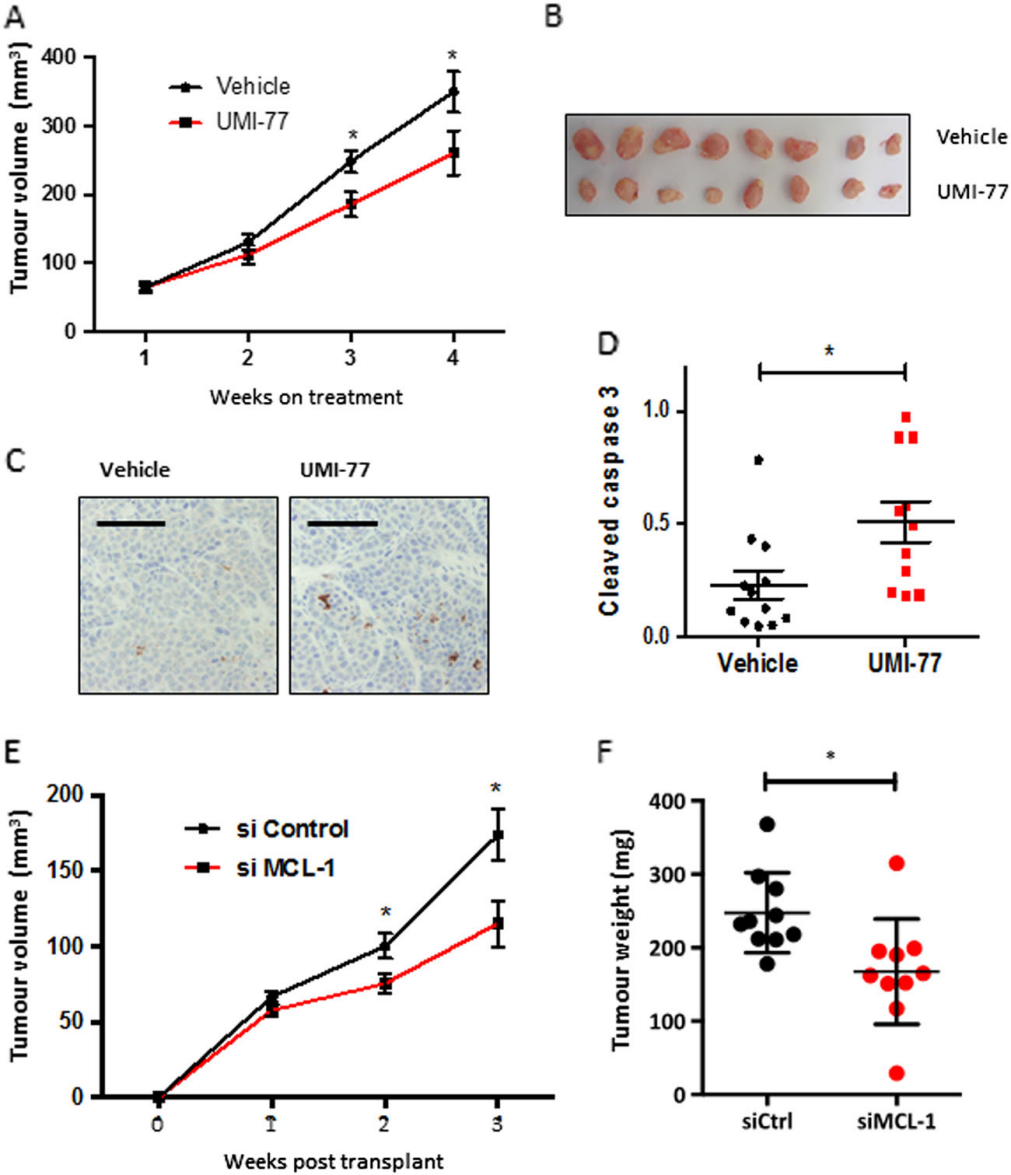
Targeting MCL-1 restricts triple-negative breast cancer cell growth* in vivo* **a** Inhibition of MDA-MB-468 tumour growth in orthologous transplantation assay in response to MCL-1 inhibitor UMI-77. Treatment commenced when tumours were ~5 mm diameter. Graph represents weekly average tumour volume ± SEM, *n* = 13 vehicle-treated (black line) and *n* = 14 UMI-77 treated (red line) *P ≤ 0.05 (Students *t*-test). **b** Representative photograph of vehicle (upper) or UMI-77 (lower)-treated tumours (as in **a**) harvested at end of experiment (after 4 weeks treatment), for weights of all tumours see Supplementary Fig. S3A. **c** Immunohistochemical analysis of cleaved caspase 3 expression in tumours harvested 4-weeks post-treatment. Representative images shown; *n* = 11–12 for each group. Scale bar is 100 μm. **d** Quantification of cleaved caspase 3 in orthologous tumours harvested 4-weeks post-treatment; bars indicate mean ± SEM and points represent average cleaved caspase 3 staining per tumour *P ≤ 0.05 (unpaired *t*-test) *n* = 12 vehicle-treated tumours and *n* = 11 UMI-77-treated tumours. Results are expressed as proportion of brown pixels (cleaved caspase 3 IHC stain) to blue pixels (nuclear stain) from 3–4 fields of view (on 10X objective) per tumour, quantified using Adobe photoshop 5.1. **e** Inhibition of MDA-MB-468 tumour growth in orthologous transplantation assay following siRNA knockdown of *MCL1*. Graph represents tumour volume from date of transplantation. Weekly average of *n* = 16 siControl (black line) and *n* = 15 *siMCL1* (red line) tumours is shown ± SEM *P ≤ 0.05 (unpaired *t*-test). **f** Reduced weight of *siMCL1* tumours harvested after 3 weeks growth *in vivo*, *n* = 10 tumours per condition harvested at this timepoint. Points indicate individual tumour weights and bars are mean ± SD *P ≤ 0.05 (unpaired *t*-test)

As treatment with MCL-1 targeting BH3-mimetic drugs induced apoptosis of breast cancer cells *in vitro* we tested the *in vivo* potential of such drugs. To clinically model breast cancer treatment we commenced pharmaceutical intervention once xenograft tumours had become clinically detectable. To this end, MDA-MB-468 breast cancer cells were injected into the mammary fat pads of BALB/c-Nude mice. Tumours were allowed to establish and when they reached ~ 5 mm diameter, treatment with the MCL-1-specific inhibitor UMI-77 (60 mg/kg) or vehicle control commenced by intraperitoneal injection 5 times per week. Treatment with UMI-77 significantly delayed growth of established MDA-MB-468 xenografts (Fig. 4a). After 4 weeks of treatment, tumours were harvested and tumour reduction confirmed and further quantified (Fig. 4b and Supplementary Fig. S3A). Low levels of apoptosis were detected in the excised tumours by immunohistochemistry for cleaved caspase 3 (Fig. 4c) while a significant increase was seen in the UMI-77-treated tumours (Fig. 4d) consistent with the on target effects of UMI-77 in MCL-1 inhibition and induction of apoptosis that we observed *in vitro* (Fig. 3d, e). To confirm this requirement for MCL-1 in breast cancer growth, a second *in vivo* approach was taken whereby MDA-MB-468 cells were treated with control siRNA or *Mcl1*-specific siRNA (Supplementary Fig. S3B) prior to injection into mammary fat pads of BALB/c-Nude mice. Tumour growth was monitored following injection and knockdown of *MCL-1* was shown to substantially impair tumour growth (Fig. 4e, f). Interestingly when end-stage tumours were harvested, MCL-1 expression had recovered (Supplementary Fig S3C, D), which reinforces that MCL-1 inhibition transiently during early tumour development can still impact on tumour growth but that selection for presence of MCL-1 ultimately occurs. These findings verify the requirement for MCL-1 in TN breast cancer growth *in vivo*.

### *Mcl1* is required for mammary tumour development *in vivo*

**Fig. 5:**
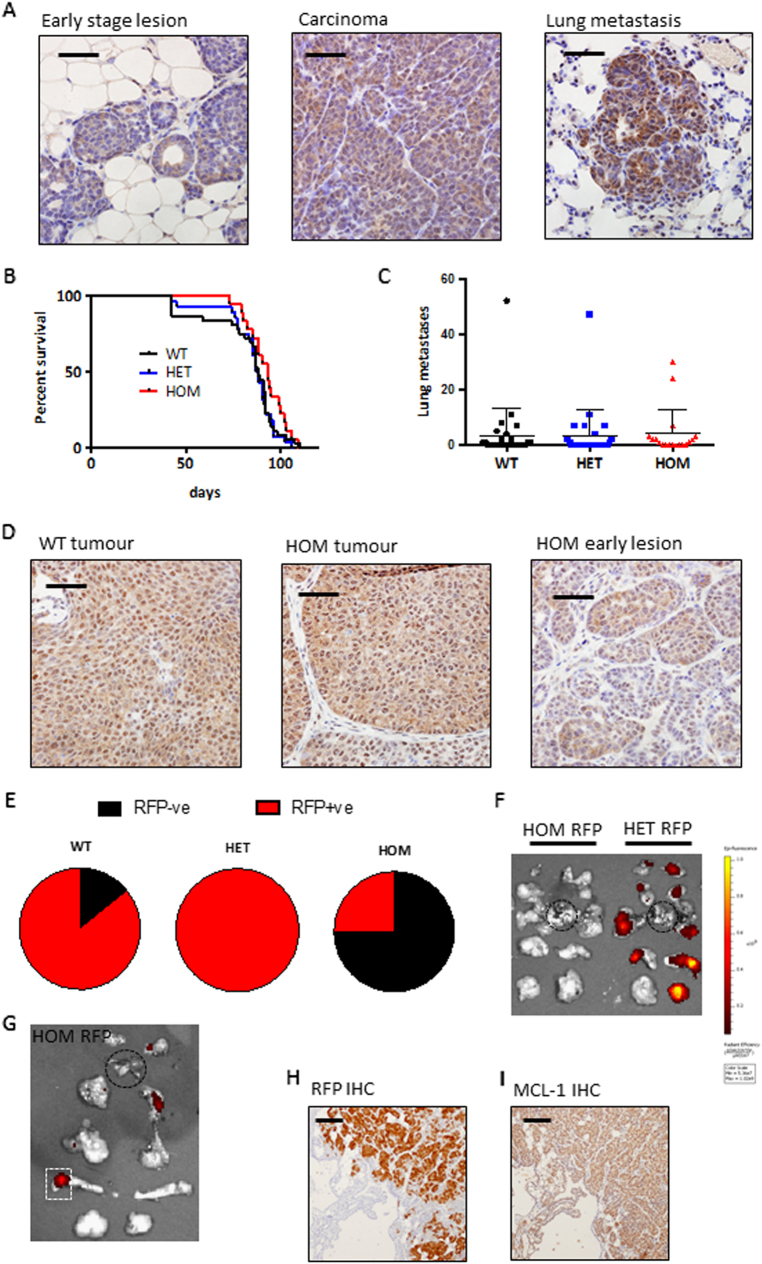
MCL-1 is required for mammary tumour development *in vivo* **a** Immunohistochemical analysis showing MCL-1 protein expression at different stages of mammary tumorigenesis (written above image) evident within a single *MMTV-PyM*T mouse. Data representative of ≥ 4 independent mice. Scale bar is 50 μm. **b** Kaplan–Meier tumour free survival analysis of *MMTV-PyMT; MMTV-Cre* transgenic female mice with targeted deletion of *Mcl1* in the mammary epithelium. Median survival of *MMTV-PyMT* mice with wild-type *Mcl1* (WT) 88 days, *n* = 36; with targeted deletion of one allele of *Mcl1* in the mammary epithelium (HET) 88 days, *n* = 28; and targeted deletion of both alleles (HOM) 93 days, *n* = 18. Full cohort and genotype information available in Supplementary Fig S4. No significant differences were observed between genotypes. **c** Microscopic lung metastases were counted in haemotoxylin and eosin stained slides of lung cross-sections from cohort mice harvested when primary tumour reached clinical endpoint. Median number of observed lung metastases was 3.4 (WT; *n* = 28), 3.3 (HET; *n* = 27), 4.2 (HOM; *n* = 18), no significant differences were observed between genotypes. Each point represents an individual cohort animal; mean and standard deviation are shown. **d** Immunohistochemical analysis of MCL-1 protein level in representative WT (left) and HOM (middle) tumours showing high levels of MCL-1 regardless of genotype while early-stage lesions in HOM mice (right) show heterogeneity in MCL-1 level. Data representative of ≥ 5 mice of each genotype/stage. Scale bar is 100 μm. **e** Pie charts showing proportion of mice with RFP-positive tumours (represented as red) as determined by IVIS imaging at clinical endpoint. WT (in 6 of 7 mice analysed); HET (in 11 of 11 mice analysed) and HOM (in 2 of 8 mice analysed). Mice scored as positive regardless of how many tumours in an individual animal scored positive. **f** Representative IVIS fluorescence imaging of *ROSA-tdRFP* reporter in mammary gland/tumours (all ten glands shown and orientated according to site of harvest with gland number 1 at top and number 5 at bottom of image) and lungs (circled in black dashed line) from HOM-RFP (*MMTV-PyMT;MMTV-cre;Mcl1*^*fl/fl*^*;ROSA-tdRFP*) and HET-RFP (*MMTV-PyMT;MMTV-cre;Mcl1*^*fl/+*^*;ROSA-tdRFP*) mice. Heat map represents fluorescence intensity with yellow being the highest. **g** Representative IVIS fluorescence imaging of *ROSA-tdRFP* reporter in a HOM-RFP mouse scored as RFP positive by IVIS imaging with minimal fluorescence staining. Orientation as described for **f**. **h**, **i** Immunohistochemical analysis of RFP expression (**h**) and MCL-1 expression (**I**) in serial sections of the small RFP-positive lesion indicated by white box in (**g**) depicting absence of MCL-1 deletion in RFP-positive cells. Scale bar is 100 μm

The associations between high MCL-1 and poor outcome, along with the observed frequent amplification of *MCL1* in breast cancer may reflect a requirement for MCL-1 in the oncogenic process as has been observed in haematopoietic cancers^32–35^. To definitively test this hypothesis we utilised a genetic mouse model of breast cancer. The *MMTV*-*PyMT* mouse recapitulates features of human breast cancer progressing through hyperplasia to metastasis^36^ in which we find high levels of MCL-1 in primary and metastatic lesions (Fig. 5a) and are able to genetically manipulate *Mcl1*. We tested the impact of reduction (heterozygous loss, HET) or deletion (homozygous loss, HOM) of MCL-1 on tumour development and metastatic spread in this model by utilising *MMTV*-*Cre* to drive specific deletion of *Mcl1*^*fl/fl*^ in the mammary epithelium of female *MMTV*-*PyMT* mice (Supplementary Fig. S4A). Mice of all genotypes were monitored for tumour development (blinded for genotype), and when tumours reached clinical endpoint, mammary tumours and lungs were harvested for analysis. Tumour related survival, number of lung metastases, and other parameters of tumour development were all comparable between mice regardless of whether mice were bred to express mammary-specific deletion of *Mcl1* or not (Fig. 5b, c and Supplementary Fig. S4). Surprisingly, immunohistochemical analysis revealed that both WT and HOM tumours expressed equally high levels of MCL-1, whereas a range of MCL-1 expression was observed in early mammary lesions in the HOM mice (Fig. 5d). These data suggest a selective pressure in mammary tumorigenesis against loss of MCL-1 and that tumour outgrowth is the result of escaper cells retaining expression of MCL-1, probably due to the *MMTV*-*Cre* not efficiently deleting the gene in all mammary cells. To address this we utilised a conditional *ROSA-tdRFP* reporter allele^37^ as a surrogate for *MMTV*-*Cre* expression. This supported our hypothesis of a selective pressure against loss of MCL-1: while 6 of 7 WT mice and 11 of 11 HET mice developed RFP-positive tumours, only 2 of 8 HOM mice developed any RFP-positive tumours (Fig. 5e, f). Furthermore, in the two HOM mice where RFP-positive tumours were detected, RFP positivity was restricted to small areas and only occurred in a minority of tumour burdened glands (see example in Fig. 5g, and compared to HET in Fig. 5f). Importantly, immunohistochemical analysis on serial sections of RFP-positive areas of tumours from HOM mice revealed equivalent levels of MCL-1 protein to neighbouring cells that were RFP negative (Fig. 5h, i). Therefore, these rare populations of tumour cells (that had tolerated Cre activation while harbouring *Mcl1*^*fl/fl*^ alleles) had emerged with MCL-1 expression intact. Altogether these data clearly indicate strong selective pressure against *Mcl1* gene loss and an absolute requirement for MCL-1 in mammary tumorigenesis.

## Discussion

Here, we report an association between high MCL-1 protein expression in tumour epithelium and poor patient outcome in breast cancer. This work significantly enhances our knowledge about the prognostic value of MCL-1 protein in breast cancer. Early studies of breast cancer patient cohorts reported opposing results when assessing MCL-1 expression; one study found no association between MCL-1 protein expression and patient outcome^38^, while another linked high MCL-1 protein with high tumour grade and poor outcome^39^. The contrasting findings of these studies (notably with smaller patient numbers of 170 and 125, respectively), with no separation by disease subtype could be explained if a prognostic role for MCL-1 occurred only in particular subsets of patients. More recently, MCL-1 was shown to be widely expressed in breast tumours, regardless of subtype or ER status^10,24^ although these studies did not report patient outcome. Intriguingly, low levels of MCL-1 protein were correlated with poor prognosis in a cohort of Luminal A breast cancer patients^24^. The same study reported differential associations between *MCL1* mRNA levels and prognosis depending on whether patients had received treatment. At the time of sampling, all of the patients in our study were treatment naive and the subsequent use of hormone therapy was almost universal across our ER + cohort. Differences in treatment exposure at time of sampling could potentially account for these disparate findings as expression of Bcl-2 family proteins, including MCL-1, are known to be altered in response to therapy^3,40^. Here, we can confirm the prognostic potential of MCL-1 at initial patient presentation.

In our study all subtypes are well represented and extensive clinicopathological data was available allowing fuller interrogation. Indeed, while we found an overall association between high MCL-1 and poor prognosis across our entire cohort (Fig. 1h), segregation of cases by receptor status (ER/HER2) revealed that although *ERBB2/HER2* amplified tumours expressed high levels of MCL-1 protein, patient outcome was not dictated by MCL-1 expression in this subtype (Fig. 2). Interestingly induction of *ERBB2/HER2* expression has been shown to increase *MCL1* mRNA levels in MCF-7 cells^41^, perhaps explaining the high level of MCL-1 protein we find in ERBB2/HER2-positive disease and it is possible that while not prognostic, these tumours could still depend on MCL-1 for survival^42^ as was indeed observed in a very recent study from the Lindeman group^23^. In our cohort, high MCL-1 protein showed similar associations with poor prognosis when cases were segregated by ER status (Fig. 2) and within the ER/PR/ERBB2-negative cohort where patients with high MCL-1 faired worst of all (Fig. 2). Intriguingly, as no relationship was found between MCL-1 protein and transcript levels in breast cancer samples^38^ it suggests to us that pathways altering MCL-1 translation or protein stability have an impact on patient survival.

In contrast to our findings with MCL-1, BCL-2 has been shown to be a favourable prognostic marker in breast cancer, often associated with slowly proliferating low grade ER-positive tumours^43–45^. High BCL-2 protein expression predicts favourable outcome regardless of ER, PR or HER2 status^46^ however, BCL2 is only expressed in a small proportion of TN breast cancers^7^ and *MCL1* mRNA is higher than *BCL2* or *BCL2L1*(BCL-XL) across all subtypes of breast cancer^10^. We find an inverse relationship between *MCL1* and *BCL2* mRNA (Fig. 1b and Supplementary Fig.  1A). It is clear that MCL-1 and BCL-2 play roles in different patient groups and that targeting MCL-1 has the potential to impact on patients with the worst prognosis, including receptor-negative breast cancer patients who currently have no targeted treatment options. Clinical trials are currently investigating ABT-199/Venetoclax in combination with tamoxifen in ER-positive metastatic breast cancer (ISRCTN98335443). It will be interesting to see whether MCL-1 levels correlate with resistance to Venetoclax in patients, as has been observed in breast cancer cell lines^10^.

The prevalence of elevated MCL-1 in breast cancer and potential for therapeutic intervention shown here and by others^10,23,24^ suggests a functional role in early tumour development. In support of this hypothesis, we found that MCL-1 expression was necessary for tumour development in the *MMTV-PyMT* mouse model of breast cancer, with outgrowth of tumours in the context of *Mcl1* deficiency only occurring when cells escaped deletion of *Mcl1* (Fig. 5). MCL-1 is known to play a role in mammary gland development, but no selection against *MMTV-cre;Mcl1*^*fl/fl*^ cells deficient for MCL-1 was observed in normal mammary gland^47^, indicating a specific dependence of mammary tumour cells on MCL-1. As the *MMTV*-*PyMT* model is regarded as a model for human luminal breast cancer^48^, our results reinforce the applicability of targeting MCL-1 in ER-positive disease where high MCL-1 correlated with poorer prognosis (Fig. 2).

In agreement with recent studies, we found that targeting MCL-1 in TN breast cancer cells; in our case using three different BH3-mimetics specific for MCL-1 (UMI-77, S63845 and A1210477); inhibited TN breast cancer cell line growth *in vitro*. Importantly, we show that this is through induction of apoptosis in a BAX/BAK and caspase-dependent manner (Fig. 3) validating the on-target effect of these drugs on mitochondrial-dependent apoptosis on breast cancer cells. To further investigate the therapeutic potential of these findings, we tested the impact of pharmacological inhibition of MCL-1 in established mammary tumours *in vivo* and found that MCL-1 inhibition or knock-down inhibited TN breast cancer growth in xenograft experiments (Fig. 4). In these models targeting MCL-1 constrained the intrinsic apoptosis pathway and inhibited TN breast cancer growth. Importantly, xenograft experiments showed that therapeutic dosing with an MCL-1 inhibitor could retard TN breast cancer growth without any apparent adverse effects on the mice. This is in line with recent studies with an additional MCL-1-specific BH3 mimetic, S63845, which showed tumour-specific cell killing in xenograft models of haematological cancers^16^.

We found that high MCL-1 protein at diagnosis predicts worse patient outcome and identifies patients that have the potential to respond to MCL-1 inhibition. MCL-1 expression has already been linked with resistance to therapy^10,40,49^. Our findings suggest this resistance may not necessarily be acquired in response to therapy, but in many cases may be innate, due to the required presence of high MCL-1 in breast cancer development. Aberrations in *MCL1* are the second most frequent genomic occurrence in treatment-resistant TN breast cancer samples^50^ and targeting MCL-1 may induce apoptosis in these tumours. Indeed in an elegant study published while this manuscript was under review, Merino et al.^23^ demonstrated the potential of MCL-1 inhibition in combination with conventional chemotherapies for increased efficacy in PDX models of TN and HER2-amplified breast cancer. This highlights the possibility of re-sensitising resistant tumours to therapy. Interestingly, additional studies provide support for this theory; in vitro experiments show cell death induction when HER2 inhibition is combined with targeting MCL-1^16^ and *in vivo* it has been shown that AZD1208 (PIM-kinase inhibitor) downregulates MCL-1 expression (among other effects) in TN breast cancer cell lines to restrict growth when used in combination with Eribulin^51^.

## Materials and methods

### Tissue microarray (TMA)

The tumour tissue microarrays were obtained from Greater Glasgow and Clyde NHS Biorepository and represent a retrospective series of primary breast cancer patients diagnosed between 1995–1998 with available clinicopathological features and outcome. The arrays were composed of 0.6 mm^3^ cores of primary operable breast tumour material from 2–3 representative areas of tumour per patient at the time of surgical resection. ER, PR and HER2 status was confirmed in cores by standard immunohistochemical/in situ hybridisation techniques.

### Immunohistochemistry

Immunohistochemistry (IHC) was carried out with antibodies to MCL-1 (Proteintech, UK), RFP (Rockland, PA, USA) or Cleaved Caspase 3 (Cell Signaling Technology, UK). Epitope retrieval was achieved by heating to 98 °C in pH-6 citrate buffer for 25 min before proceeding as per the manufacturers instructions with MCL-1 antibody used at a dilution of 1:300 and Cleaved Caspase 3 antibody at 1:500. IHC for cleaved caspase 3 was quantified with Adobe photoshop 5.1 using the method described by Lehr and colleagues^52^.

### TMA scoring

MCL-1 immunoreactivity in the cytoplasm of tumour epithelium was quantified using a weighted histoscore method to give a value of 0–300 by K.J.C.^31^ Ten percent of total core number was scored by two observers (K.J.C. and N.F.) independently and blind to the other observers score. Inter-observer agreement was measured by Interclass Correlation Coefficient. All scoring was performed blind to clinical and pathological data, and cutoff for MCL-1 high vs. low was set at histoscore of 168 to cut the group equally without splitting cases with identical scores into different MCL-1 groups.

### Statistical analysis

Statistical significance between experimental groups, *P* < 0.05, was calculated by Unpaired *t*-test, two tailed, with GraphPad Prism version 6.0c (Graphpad Software, CA, USA). Kaplan–Meier survival curves of breast cancer-specific survival were also plotted using GraphPad Prism version 6.0c and Mantel-Cox (Log-rank) analysis used to determine significant differences in survival. For associations between MCL-1 expression and clinical parameters Pearson Chi-Square tests were performed using SPSS software version 19 (IBM Corp, NY, USA).

### Genetically engineered mouse models

Animals were housed in a barriered facility proactive in environmental enrichment. All work was carried out in line with the Animals (Scientific Procedures) Act 1986 and the EU Directive 2010 and was sanctioned by the local ethical review process (University of Glasgow). *MMTV-Cre* (kindly supplied from WJ Muller, McGill University)*, MMTV-PyMT* (The Jackson laboratory, ME, USA), *Mcl1*^*tm3Sjk*^ *(Mcl1* ^*fl/fl*)^(The Jackson laboratory, ME, USA), and *ROSA-tdRFP* mice (acquired from the European Mouse Mutant Archive (EMMA)) have all been described previously^37,53–55^. All mice had been back-crossed > 7 generations FVB/N and all controls were littermates. Mice were monitored 2–3 times per week for tumour development, onset was defined when the first tumour was detectable at 5 mm diameter and clinical endpoint at 15 mm diameter. At endpoint, mice were sacrificed; mammary gland/tumours and lungs were excised and imaged using the IVIS Spectrum imaging system (PerkinElmer, MA, USA). Images were analysed using the IVIS Living Image software. Organs were weighed before fixation in 10% formalin and embedding in paraffin wax. Microscopic metastases were detected in haemotoxylin and eosin stained cross-sections of lungs under 10X magnification.

### Human cell lines

Human cell lines were originally sourced from the American Type Culture Collection (ATCC) and were authenticated by Promega GenePrint 10 System (Promega WI, USA). Cells were maintained at 37 °C with 5% CO2 with 10% fetal bovine serum (FBS), except for *in vitro* experiments using A1210477 where FBS level was reduced to 3% during drug treatment and also in the relevant control samples. Cell viability was determined by CellTiter 96 MTS assay (Promega) after 48 h incubation with the indicated concentration of MCL-1 inhibitor UMI-77 (Selleck, UK), S63845 (Apexbio, UK) or A1210477 (Apexbio, UK). SYTOX Green (Invitrogen, UK) was used to identify dead cells and cell confluence measured using the Incucyte Live Cell Analysis System (Essen Bioscience, UK). 10 μM etoposide (Sigma, UK) was used to induce apoptosis and 10 μM Q-VD-OPh (Apexbio, UK) was used to block caspase activity. CRISPR/Cas9 gene editing using the LentiCRISPRv2 system (Addgene, MA, USA) was performed for BAX and BAK as described previously^2^.

### Western blotting

Standard western blot procedures were used on whole cell lysates and probed with antibodies specific to MCL-1 (Proteintech, UK), ACTIN (Sigma, UK), PARP (Cell Signaling, UK), BAK (Cell Signaling, UK), BAX (Santa Cruz, CA, USA), HSP70 (Cell Signaling, UK), Active Caspase 3 (Cell Signaling, UK).

### Xenograft experiments

For assessment of UMI-77 anti-tumour activity *in vivo,* 3 million MDA-MB-468 breast cancer cells were injected bilaterally into the inguinal mammary fat pads in 1:1 PBS:matrigel mix into 8-week BALB/c-Nu female mice (Charles River, UK). Treatment commenced 2 weeks after injection and UMI-77 was administered by intraperitoneal injection at 60 mg/kg in a regime of 5 daily doses followed by 2 rest days. For *in vivo* use UMI-77 was dissolved in 5% DMSO/30% PEG300/ 65% dd H_2_0. Tumour growth was monitored by caliper measurement three times per week and volume calculated using the equation ([length × width^2^]2). Graphs represent average of three weekly measurements relative to tumour volume at commencement of treatment. Tumours were harvested after 4 weeks of treatment.

MCL-1 knockdown was achieved using a pool of prevalidated siRNA to human MCL-1 s8583 (Ambion/Life Technologies, UK) at 5 nM concentration or non-targeting control siRNA and nucleofection using Amaxa kit (Lonza, UK) according to the manufacturer's protocol. For orthologous transplantation assay of siRNA-treated MDA-MB-468 cells, 3 million siMcl1 or siSCR treated cells in a 1:1 PBS:matrigel mix were injected bilaterally into the inguinal mammary fat pads of 6 week female BALB/c-Nu mice (Charles River, UK) 18 h after nucleofection.

## Electronic supplementary material

Supplementary Figure Legends

Supplementary Figures
