# Supplementary material for: MCL-1 is a prognostic indicator and drug target in breast cancer
Source: Cell Death Dis. 2018 Jan 16;9(2):19. doi: 10.1038/s41419-017-0035-2 (PMC5833338; doi:10.1038/s41419-017-0035-2)
Supplement: Supplementary file 1 — Supplementary Figure Legends [file 41419_2017_35_MOESM1_ESM.docx]

Campbell et al, MCL-1 in breast cancer

**Supplementary figure legends**

**Supplementary Figure S1**

Scatterplots of *MCL1* mRNA expression versus *BCL2, BCL2L1* (BCL-XL), *BCL2A1* and *BCL2L2* (BCL-W) in METABRIC dataset of 2999 breast tumours (28). p<0.05 R>0.04.

**Supplementary Figure S2**

**A.** MTS assay showing viability of MCF7 cells after 48hr treatment with indicated dose of MCL-1 inhibitor UMI-77. Bars indicated mean ±SD, of n=5 independent experiments plated in triplicate.

**B.** Western blot analysis of MDA-MB-468 cells CRISPR/Cas9 engineered for BAX/BAK double knockout or non-targeting control.

**Supplementary Figure S3**

**A.** UMI-77 restricts MDA-MB-468 tumour growth in orthologous xenotransplant assay. Graph indicates mean tumour weight ±SD after 4 weeks treatment, n=13 Vehicle treated tumours and n=14 UMI-77 treated tumours *P≤0.05 students t test. As per Figure 4B.

**B**. Western blot showing MCL-1 knockdown in MDA-MB-468 cells 72h following siRNA treatment.

C. Western blot showing recovery of MCL-1 expression in tumours harvested at endpoint. Each lane represents tumour harvested from independent mice, n=3 per condition.

D. Immunohistochemical analysis of MCL-1 protein expression in tumours harvested at endpoint, representative of n=3 tumours per condition. Independent mice to those shown in Fig S3C. Scale bar 100 μm.

**Supplementary Figure S4**

**A.** Description of mouse genotypes with n numbers (all female) used in *MMTV-PyMT* mammary tumorigenesis studies in Figure 5 and Supplementary Figure 5B.

**B-E** Supplementary to Figure 5: further details for *MMTV-PyMT* cohort mice. **B** Number of days from birth until first palpable tumour (5mm). **C** Time taken for tumour growth from palpable tumour (5mm), until clinical endpoint (15mm). **D** Number of tumour burdened glands at clinical endpoint (out of 10 possible). **E** Total mammary gland weight at endpoint. Points indicate individual mice and bars are mean ±SD. No significant differences were detected between genotypes, WT; n=36, HET; n=28, HOM; n=18.
