# Supplementary material for: MCL-1 is a prognostic indicator and drug target in breast cancer
Source: Cell Death Dis. 2018 Jan 16;9(2):19. doi: 10.1038/s41419-017-0035-2 (PMC5833338; doi:10.1038/s41419-017-0035-2)
Supplement: Supplementary file 2 — Supplementary Figures [file 41419_2017_35_MOESM2_ESM.pptx]

## Slide 1
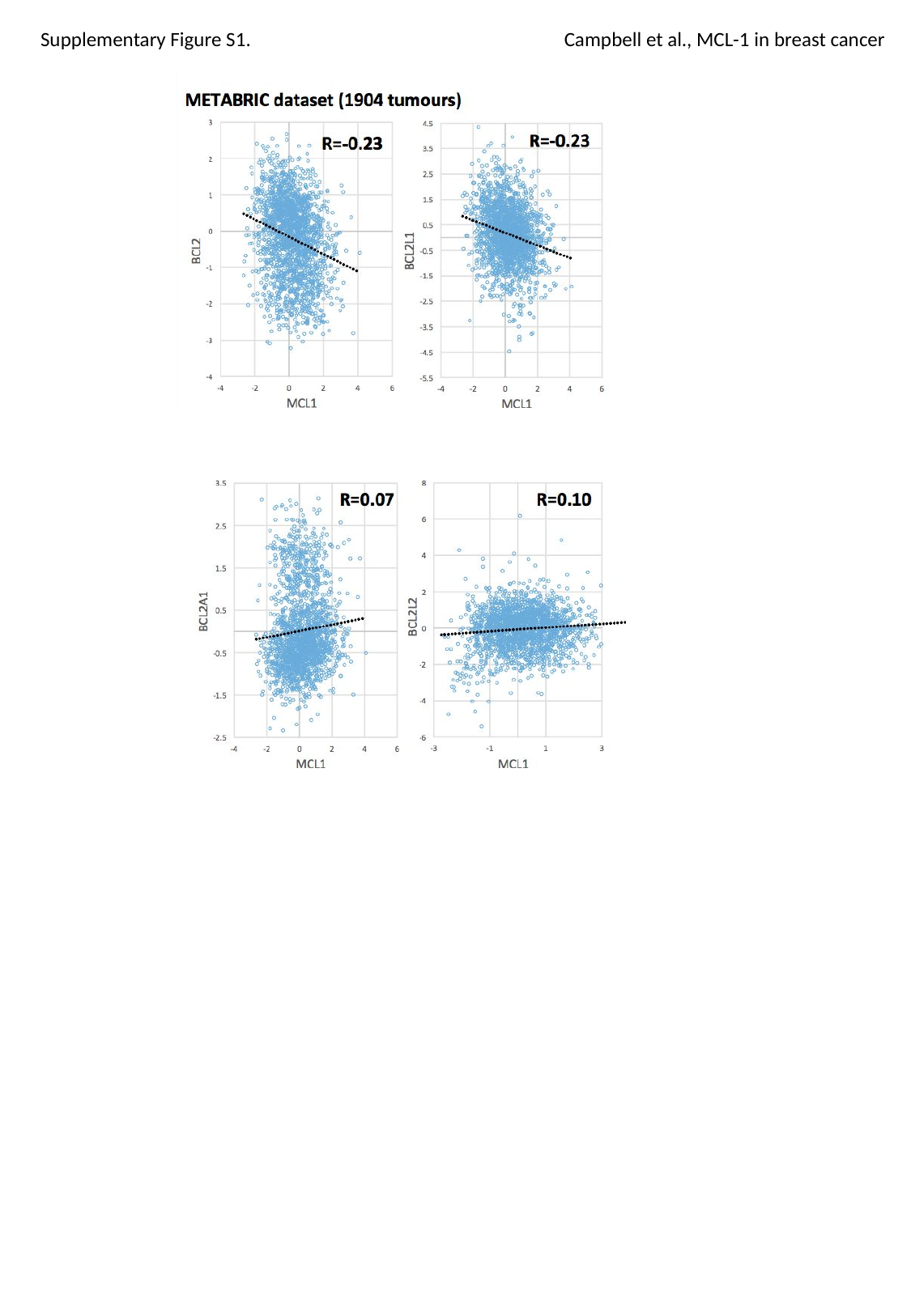

Supplementary Figure S1.
Campbell et al., MCL-1 in breast cancer

## Slide 2
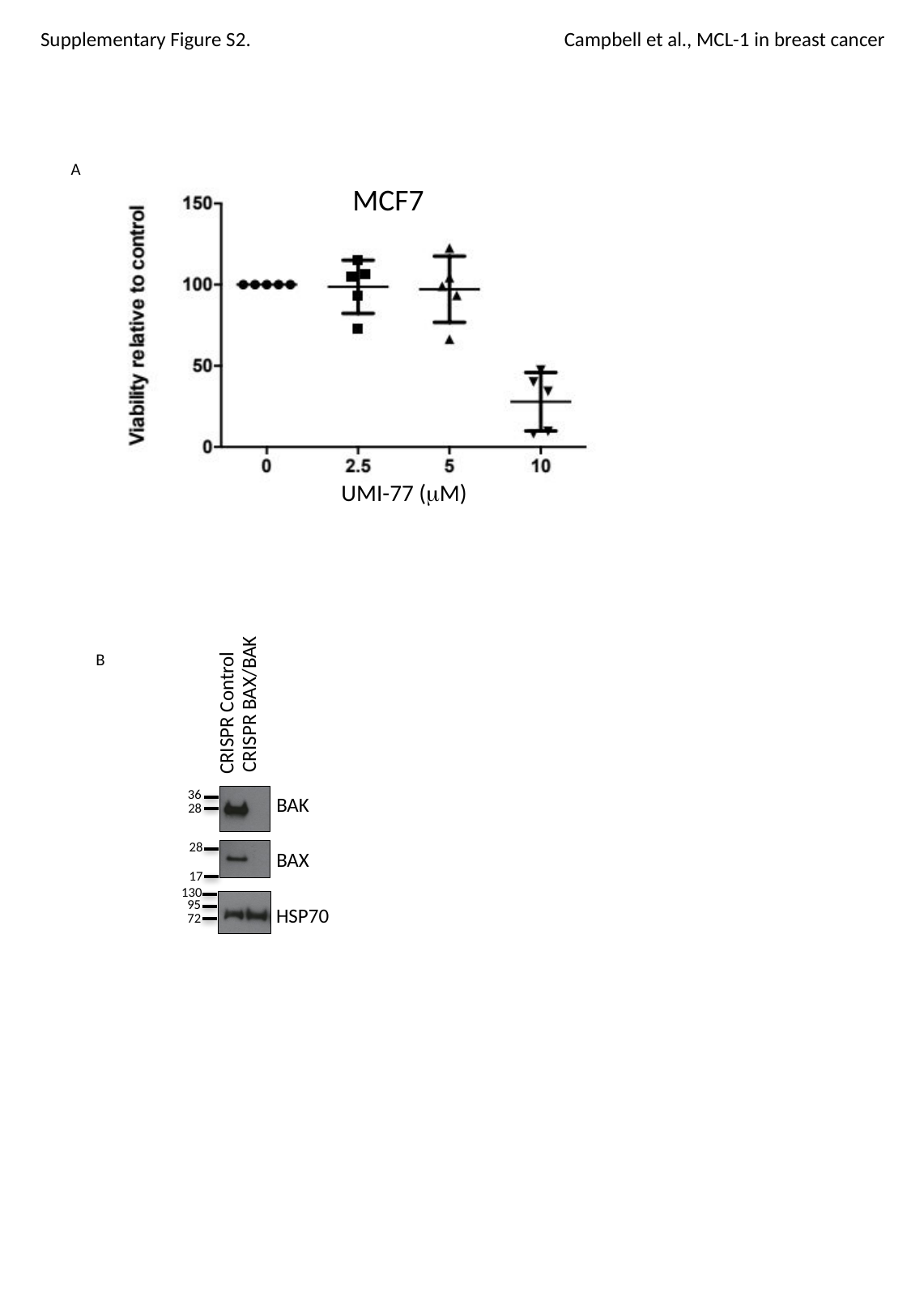

Supplementary Figure S2.
Campbell et al., MCL-1 in breast cancer
A
MCF7
UMI-77 (mM)
CRISPR BAX/BAK
B
CRISPR Control
36
BAK
28
28
BAX
17
130
95
HSP70
72

## Slide 3
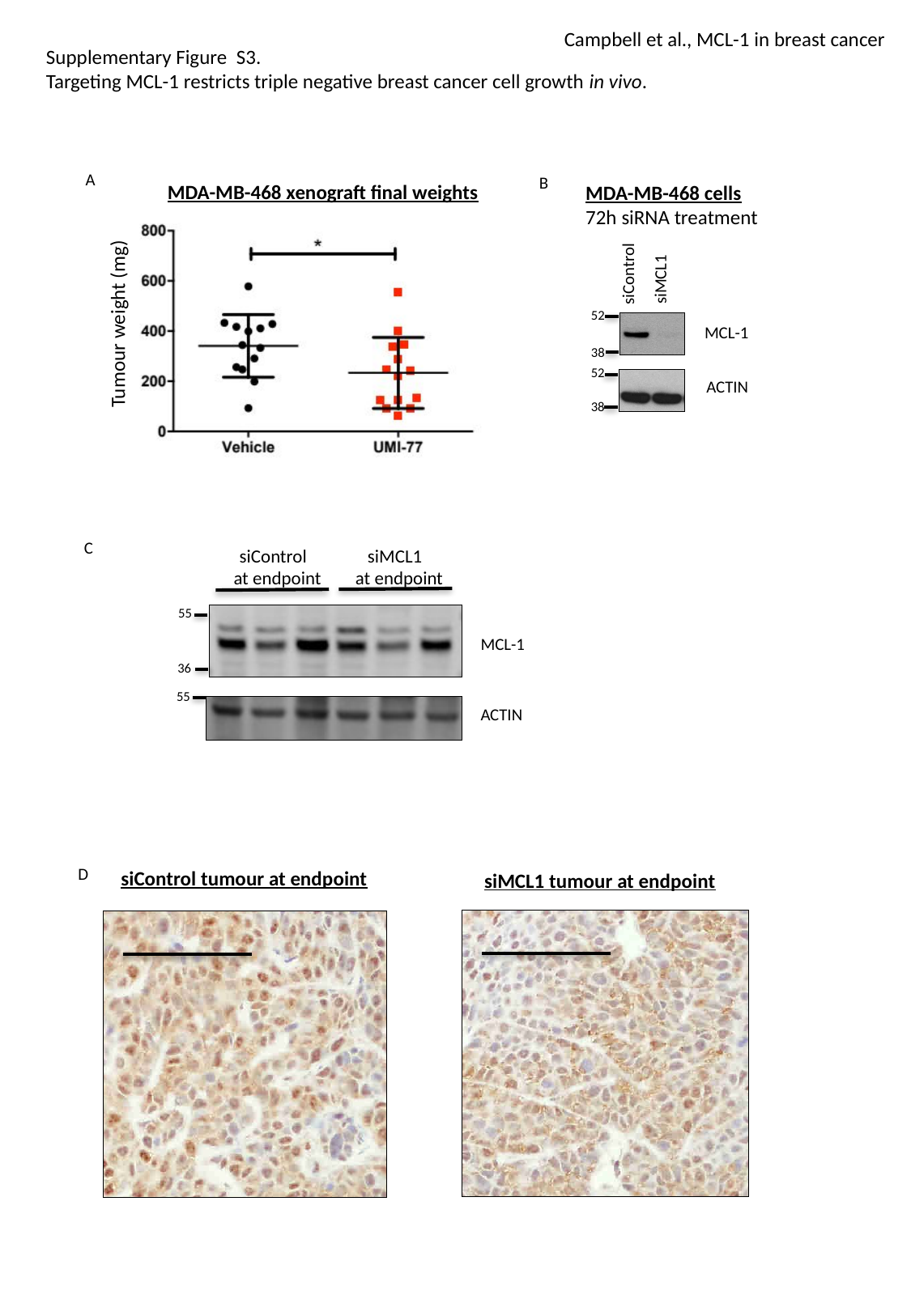

Campbell et al., MCL-1 in breast cancer
Supplementary Figure S3.
Targeting MCL-1 restricts triple negative breast cancer cell growth in vivo.
A
B
MDA-MB-468 xenograft final weights
MDA-MB-468 cells
72h siRNA treatment
siControl
siMCL1
52
Tumour weight (mg)
MCL-1
38
52
ACTIN
38
C
siControl
 at endpoint
siMCL1
 at endpoint
55
MCL-1
36
55
ACTIN
D
siControl tumour at endpoint
siMCL1 tumour at endpoint

## Slide 4
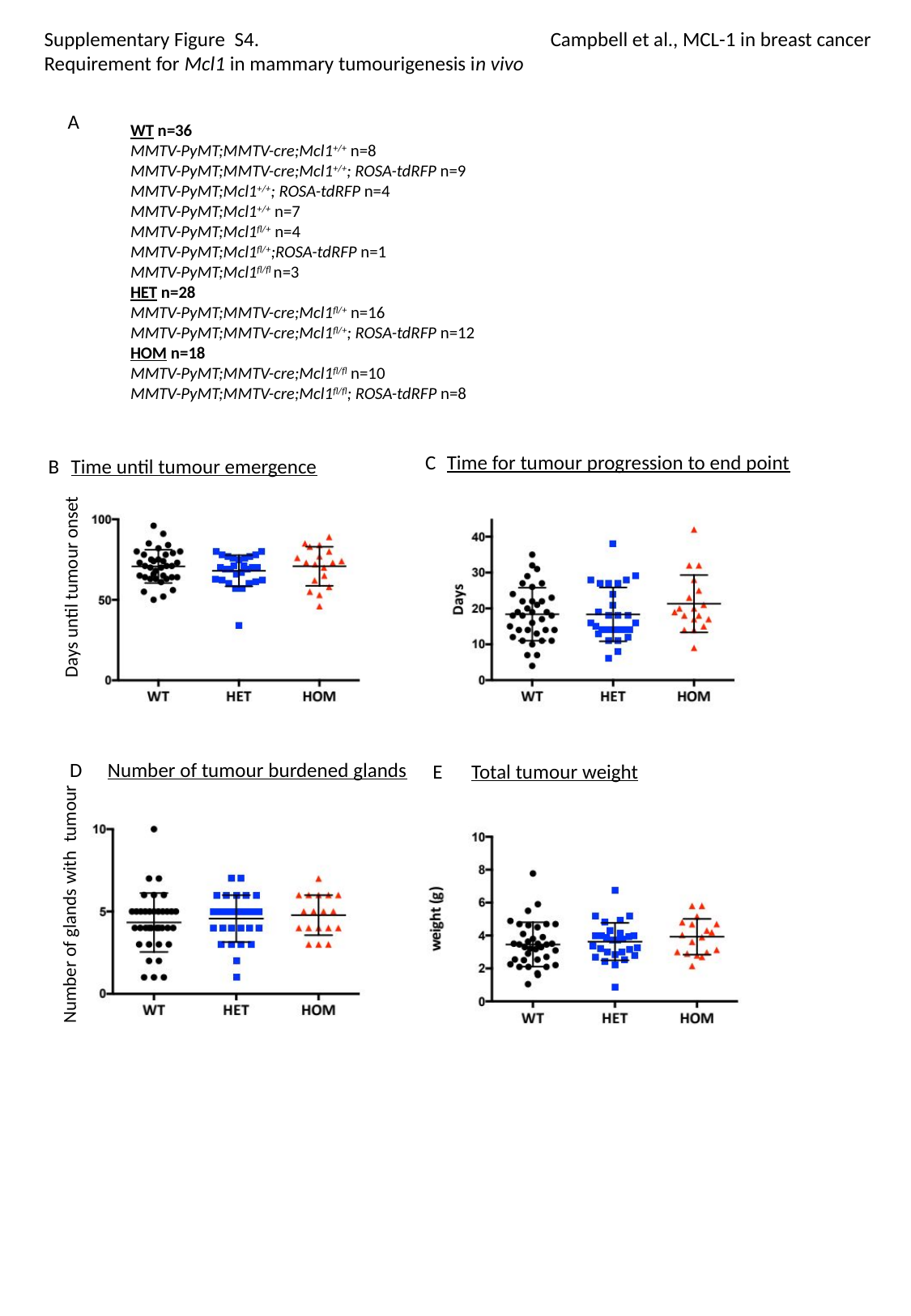

Supplementary Figure S4.
Requirement for Mcl1 in mammary tumourigenesis in vivo
Campbell et al., MCL-1 in breast cancer
A
WT n=36
MMTV-PyMT;MMTV-cre;Mcl1+/+ n=8
MMTV-PyMT;MMTV-cre;Mcl1+/+; ROSA-tdRFP n=9
MMTV-PyMT;Mcl1+/+; ROSA-tdRFP n=4
MMTV-PyMT;Mcl1+/+ n=7
MMTV-PyMT;Mcl1fl/+ n=4
MMTV-PyMT;Mcl1fl/+;ROSA-tdRFP n=1
MMTV-PyMT;Mcl1fl/fl n=3
HET n=28
MMTV-PyMT;MMTV-cre;Mcl1fl/+ n=16
MMTV-PyMT;MMTV-cre;Mcl1fl/+; ROSA-tdRFP n=12
HOM n=18
MMTV-PyMT;MMTV-cre;Mcl1fl/fl n=10
MMTV-PyMT;MMTV-cre;Mcl1fl/fl; ROSA-tdRFP n=8
C
Time for tumour progression to end point
B
Time until tumour emergence
Days until tumour onset
D
Number of tumour burdened glands
E
Total tumour weight
Number of glands with tumour
